# Dual-atria rotor modification: A comparative analysis of rotor modification and posterior wall isolation in patients with persistent and long-standing persistent atrial fibrillation

**DOI:** 10.1016/j.hroo.2025.05.021

**Published:** 2025-05-27

**Authors:** Yoshifumi Okano, Hironobu Tanii, Katsuya Akitsu, Yamato Mifune, Kosuke Takeda, Yuichi Moriyama, Hosei Kikushima, Shintaro Yao, Masaya Shinohara, Hirotsugu Matsumoto, Kazuhito Suzuki

**Affiliations:** 1 Division of Cardiology, Tokyo Metropolitan Ohkubo Hospital, Shinjyuku-ku, Tokyo, Japan; 2 Division of Cardiovascular Medicine, Toho University Omori Medical Center, Ohta-ku, Tokyo, Japan; 3 Department of Radiology, Tokyo Metropolitan Ohkubo Hospital, Shinjyuku-ku, Tokyo, Japan

**Keywords:** Persistent atrial fibrillation, Phase mapping, Posterior wall isolation, Right atrium, Rotor modification, Spiral waves, Wavefront dynamics

## Abstract

**Background:**

Various methods have been devised for catheter ablation of persistent atrial fibrillation (AF). However, it remains difficult to understand the mechanism of AF and to determine the optimal method.

**Objective:**

This study aimed to evaluate the effectiveness of rotor modification (RM) compared to posterior wall isolation (PWI) in the treatment of persistent AF.

**Methods:**

The study included 50 patients in the RM group (mean age: 67.0 ± 8.7 years; 76% with long-standing persistent AF) and 50 patients in the PWI group (mean age: 66.8 ± 8.9 years; 66%). In the RM group, in addition to cryoballoon isolation of the pulmonary veins, rotors in both atria were identified using a phase mapping system and ablated at low power. In the PWI group, the pulmonary vein antrum and posterior wall were isolated together using a radiofrequency catheter. The primary end point was the maintenance of sinus rhythm, defined as freedom from atrial tachycardia, AF, and atrial flutter over a 36-month postoperative follow-up period.

**Results:**

Survival curve analysis using the log-rank test revealed a statistically significant difference (*P* < .001), demonstrating the superiority of RM.

**Conclusion:**

RM is suggested to be as effective as PWI or more effective in maintaining sinus rhythm in patients with persistent AF.

**Key Findings**

▪ This study compared rotor modification with posterior wall isolation for the treatment of persistent atrial fibrillation.
▪ Rotor modification, which included phase mapping and targeted low-power ablation of sustained rotors in both atria, resulted in significantly higher sinus rhythm maintenance over a 36-month follow-up period compared to posterior wall isolation (*P* < .001).
▪ Rotor activity was observed with similar frequency in the left and right atria, particularly near anatomically complex regions, highlighting the potential value of biatrial mapping and intervention.
▪ The findings suggest that rotor-guided ablation is a promising and possibly superior alternative to conventional posterior wall isolation for persistent atrial fibrillation.
▪ Further large-scale studies are warranted to confirm the efficacy of this targeted, low-power biatrial approach.

## Introduction

Atrial fibrillation (AF) is the most prevalent arrhythmia encountered in clinical practice, with its incidence increasing with age. It is a major cause of cardiogenic embolism and reduced exercise tolerance. Persistent AF (PeAF) is associated with heart failure and left atrial (LA) dilation.^1^^,^^2^ Although treatment options such as anti-coagulation, rate control therapy and anti-arrhythmic drugs are available, their efficacy is often limited. As a nonpharmacological intervention, various ablation methods have been developed, including pulmonary vein isolation (PVI) alone,^3^ posterior wall isolation (PWI),^4^, ^5^, ^6^, ^7^ appendage ablation,^8^ Marshall ablation,^9^ rotor ablation,^10^, ^11^, ^12^, ^13^, ^14^ and various ablation and mapping methods have been reported.^15^, ^16^, ^17^, ^18^ Among these, PVI and PWI have been extensively studied with several procedural approaches to PWI, including BOX isolation (roof and floor line ablation), balloon-based isolation, and the hybrid convergent procedure. Rotor ablation has been proposed as a complementary strategy to these isolation procedures. In this study, we investigated the effectiveness of rotor modification (RM) using a phase mapping system. This novel approach involves visually classifying the wavefront dynamics of the spiral waves observed in color phase movies, allowing for precise identification of rotors in both atria. Low-power radiofrequency (RF) ablation was applied to these identified rotors. To assess the effectiveness of RM, we conducted a long-term comparative analysis against PWI.

## Methods

The main objective of this study is to compare the clinical results (freedom from atrial tachycardia [AT], AF, atrial flutter [AFL], or the occurrence of complications) in the 2 treatment groups. A retrospective cohort study was conducted on patients who underwent PWI or RM (PeAF and long-term PeAF). The PWI group included 50 patients treated between 2018 and 2020. The RM group included 50 patients treated between 2020 and 2021. The cases included in this study are limited to those undergoing PVI for the first time. Long-standing persistent AF was defined as AF lasting for 12 months or longer. Patient characteristics such as age, gender, B-type natriuretic peptide levels, left ventricular ejection fraction, and CHA_2_DS_2_-VASc score are summarized in Table 1. The primary end point of this study was defined as the freedom from AT/AF/AFL. Data were analyzed using Kaplan-Meier curves and Cox proportional hazard models. The presence or absence of AT/AF/AFL was determined by 12-lead electrocardiograms (ECGs) performed at 3-month intervals and Holter ECG recordings performed at 3, 6, 12, 18, 24, 30, and 36 months after the procedure. These objective findings were further evaluated in conjunction with a comprehensive assessment of the patient’s symptoms. There are significant differences between the PWI and RM groups in terms of the devices used for PVI. In Japan, the use of cryoballoon for PeAF was approved in 2020. In addition, there is a report that the BOX isolation for PeAF reduces the number of rotors in the LA. Against this background, we evaluated the usefulness of RM itself using a cryoballoon without performing extended PV antrum isolation. The impact of this difference in technique on long-term outcomes cannot be ignored.

**Table 1:** Patient characteristics

	RM group (n = 50)	PWI group (n = 50)	*P* value
Age, mean (SD), years	67.0 (8.7)	66.8 (8.9)	.883
Male (%)	35 (70)	37 (74)	.714
BMI, mean (SD), kg/m^2^	25.0 (4.2)	24.5 (4.2)	.581
BSA, mean (SD), m^2^	1.69 (0.30)	1.68 (0.19)	.752
BNP, median (25, 75%), pg/mL	92.8 (53.1, 140.5)	98.1 (58.5, 135.2)	0.742
CHA2D2-VASCs score, mean (SD), pts	3.7 (1.4)	3.8 (1.8)	0.399
CHADS2 score, mean (SD), pts	2.3 (1.1)	2.5 (0.8)	0.344
LVEF, mean (SD), %	59.1 (13.6)	58.2 (9.6)	.238
LAD, mean (SD), mm	42.9 (7.3)	44.6 (6.2)	.434
Long-standing PeAF (%)	38 (76)	33 (66)	.378
Anti-arrhythmic drug			
Class I	33	32	1.00∗
Class III	10	8	1.00∗
Class IV	7	10	1.00∗
Procedure time, mean (SD), minutes	186.9 (43.2)	215.7 (56.0)	.023†
Number of complications, (n)			
Hematoma	2	2	
Pseudo aneurysm	1	0	
Cardiac tamponade	0	1	
Arrhythmia induced by AF induction			
Mitral flutter	3	7	
Common atrial flutter	6	8	
Non-PV foci and other	5 (SVC = 1)	4	

AF = atrial fibrillation; BMI = body mass index; BSA = body surface area; BNP = B-type natriuretic peptide; LVEF = left ventricular ejection fraction; LAD = left atrial dimension; PeAF = persistent atrial fibrillation; PV = pulmonary vein.

∗ Fisher exact test (2-sided)

† Significant difference at <.05.

### Rotor Modification

**Figure 1:**
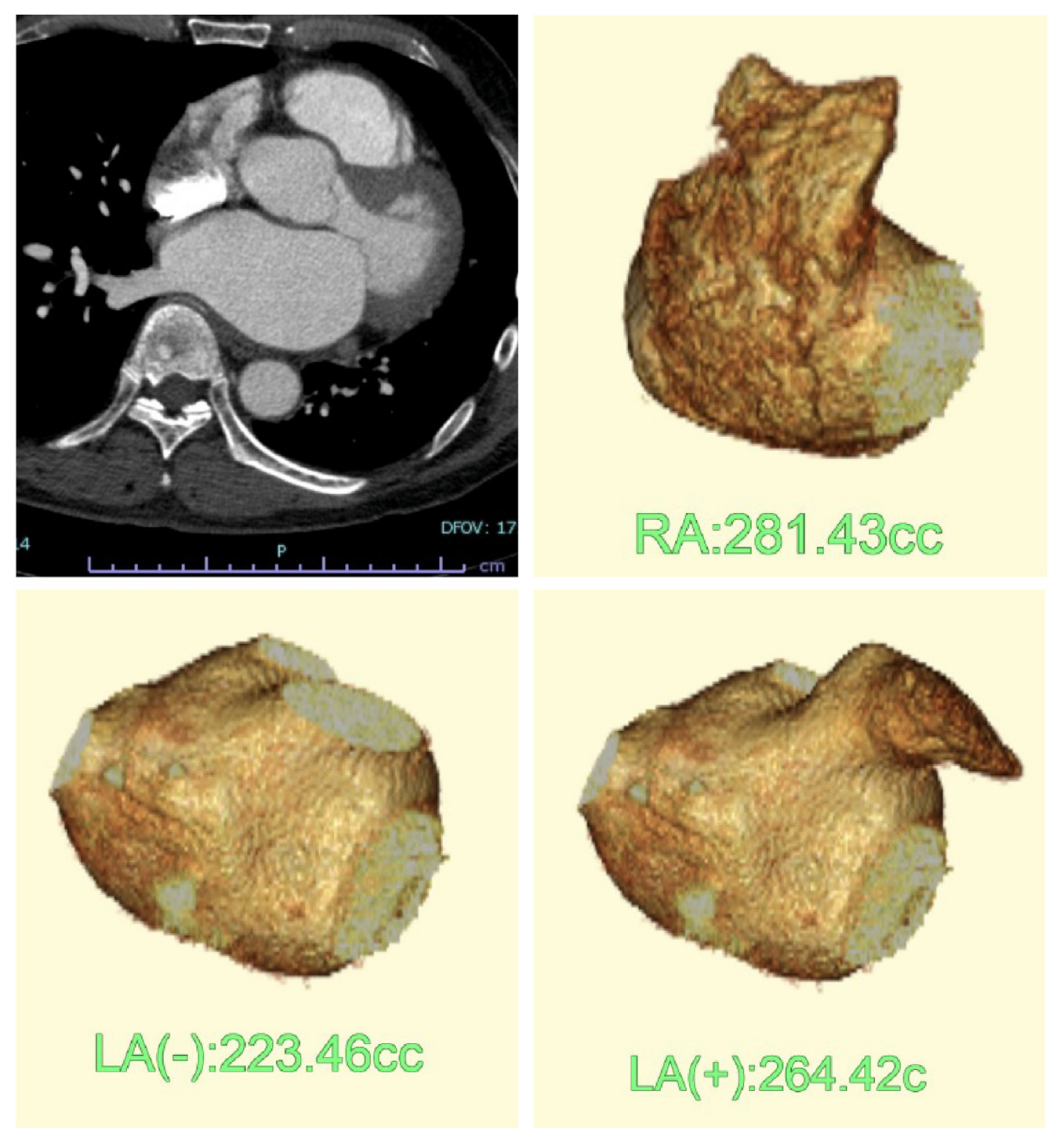
The figure shows the method used to calculate the atrial volume using diagnostic imaging software. The *top left* section shows the original image obtained from a contrast-enhanced computed tomography scan. The software processes the image, reconstructing it into standard voxels and calculating the internal volume. The *top right* figure shows a visual representation of the right atrium. There is a considerable individual variation in the volume of the left appendage. This individual variation is very important because it can affect the results of this study. For this reason, we have adopted 2 volume indices for comparative analysis. The diagram on the *lower left* shows the left atrium without appendage, and the diagram on the *lower right* shows the left atrium with appendage.

**Figure 2:**
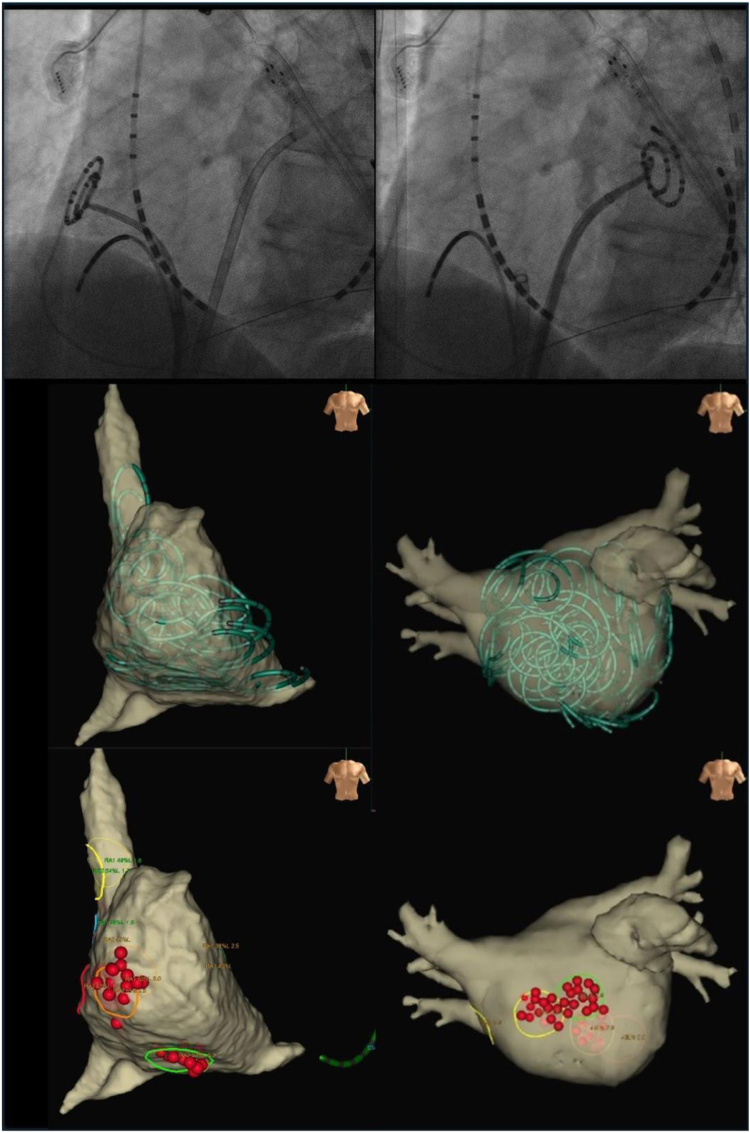
The figures show an example of phase mapping and low-power radiofrequency ablation. The 2 images in the upper row are fluoroscopic (left anterior oblique views) showing the insertion of a spiral electrode catheter into the atrium, placement of a 20-pole electrode catheter into the coronary sinus, and placement of an 8-pole electrode catheter in the right atrium. The image on the left shows the mapping of the right atrium, and the image on the right shows the mapping position in the left atrium. The image below shows the mapping location of the regions of interest (ROI) displayed on the 3D electro-anatomical model of the EnSite system. The *green electrodes* on the EnSite system indicate the location of the spiral electrodes, and the mapping location is recorded with a number. The target-ROI for rotor modification is selected by visually analyzing the color phase movies of each ROI. The location where the ablation was performed is indicated with a *red tag*.

**Figure 3:**
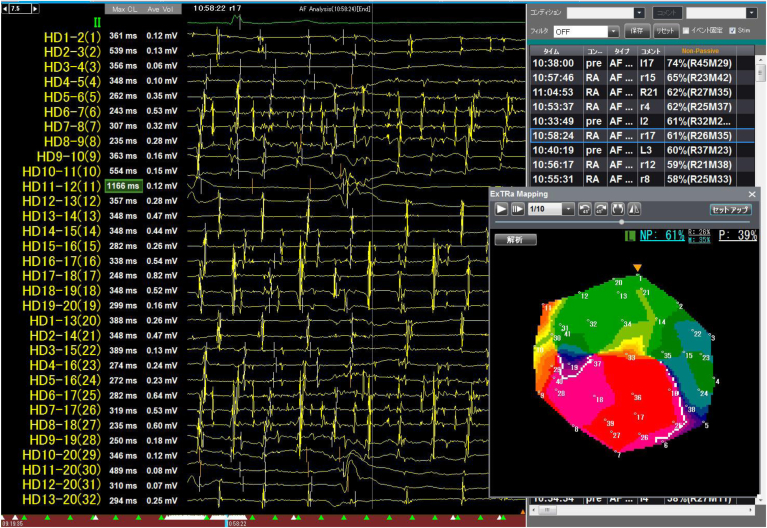
The figure shows the analysis screen for phase mapping using the EXT system. On the left side of the screen, a real-time intra-cardiac electrogram is displayed. This shows the bipolar electrogram obtained from a single region of interest (41-poles) using a spiral electrode. On the lower right of the screen, a color phase movie is displayed. The positions of the electrodes related to the intra-cardiac electrogram are indicated by numerical labels in the movie. The depolarization process at each electrode position is indicated by a change in color from *red* to *purple*. This process is also explained in Figures 4 and 5.

**Figure 4:**
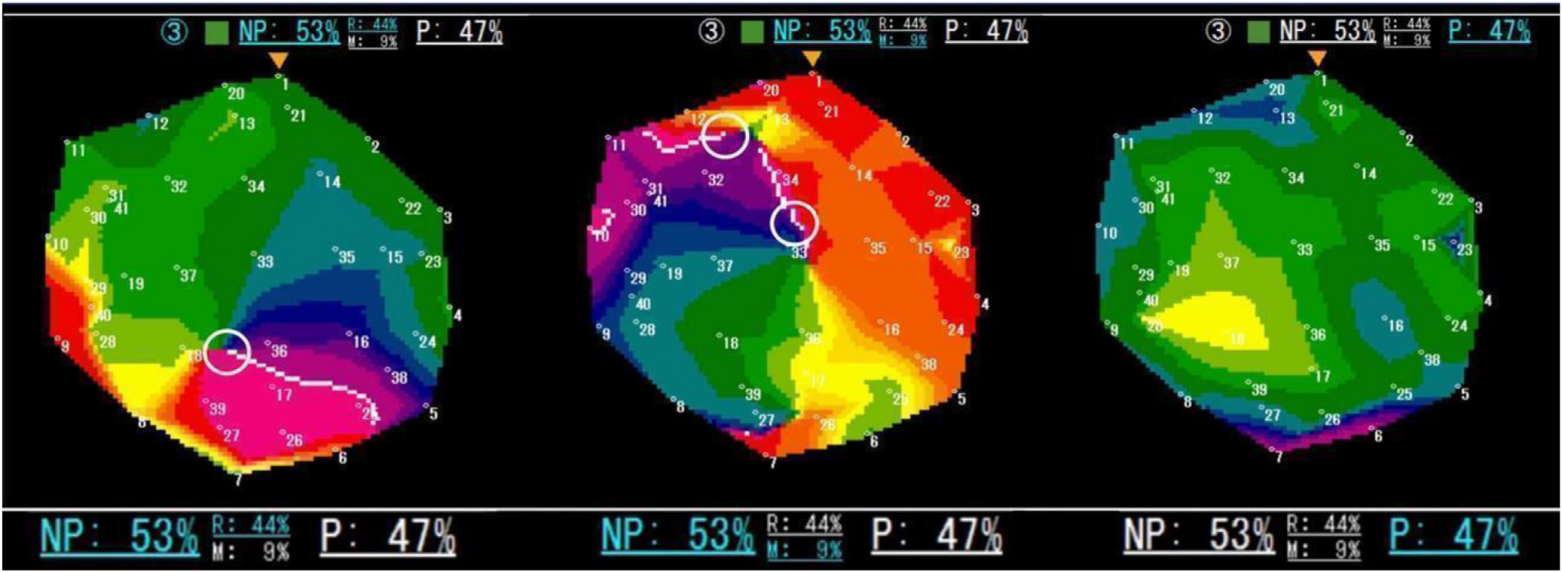
This figure shows the actual color phase movie of the EXT system. The EXT system employs artificial intelligence and deep learning to analyze the electrical activity associated with atrial fibrillation. Based on mapping recordings, the system automatically classifies activation patterns as follows: passive activation (P), non-passive activation (NP), and spiral waves present, which can be further divided into single spiral wave reentry (“R”) and multiple wavelets (“M”). The proportion of NP relative to the total recording time is expressed as %NP. In this specific region of interest, the system measured as follows: NP, 53%; R, 44%; M, 9%; P, 47%. The indicators displayed in *blue* highlight the rotor status at a given moment. Left image (t = 240 ms/5000 ms): the *white frame* (added for illustration) marks a phase singularity, indicating a single spiral wave rotation. EXT system classifies this as “R,” and both “NP” and “R” are highlighted in *blue*. Middle image (t = 2400 ms/5000 ms): Two spiral waves are detected, leading to classification as “M.” Both “NP” and “M” are displayed in *blue*. Right image (t = 3200 ms/5000 ms): No phase singularities (core) or spiral waves detected, indicating passive activation “P.” “P” is highlighted in *blue*. These 3 consecutive images are snapshots taken within region of interest. Since “NP” and “P” appear intermittently throughout the 5-second observation period, their proportions are quantified over time. The lower section of the figure provides a magnification for enhanced clarity.

**Figure 5:**
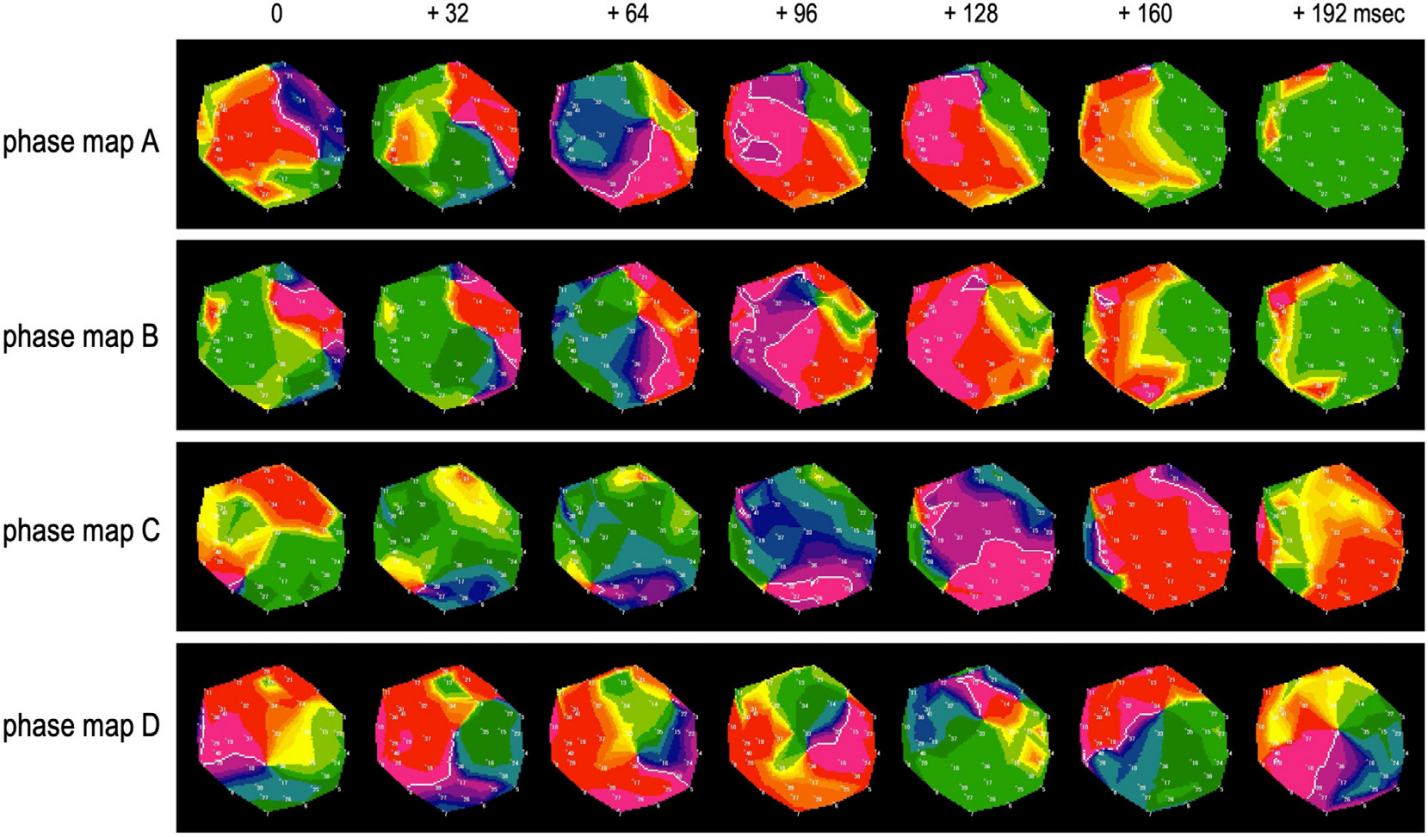
This figure shows snapshots of a typical rotor observed using a color phase movie from the EXT. The EXT system generates data for the region of interest (ROI) at a rate of 250 frames per second (4 ms/f). This figure shows snapshots of the color phase movie displayed from left to right at 32 ms intervals. Phase map A shows an example where the wavefront has not rotated sufficiently, and the rotor duration is extremely short. In the 0 ms snapshot, the phase singularity (core) is at 3 o’clock in the ROI, and the wavefront is observed in the 12 o’clock direction. After that, the wavefront rotates clockwise, but in the 160 ms snapshot, wavebreak is observed. Phase map B shows snapshots of the case where 2 rotors are observed within a single ROI. In the 0 ms snapshot, 2 rotors with phase singularities (cores) are observed at 12 o’clock and 3 o’clock. The wavefront of the 12 o’clock rotor rotates counterclockwise, while the wavefront of the 3 o’clock rotor rotates clockwise. These 2 wavefronts collide after 128 ms, and the rotors disappear or wavebreak. In the EXT, even in the absence of stable rotation, as seen in these 2 examples, it is classified as non-passive activation due to the presence of multiple wavelets. However, in this study, the ROI was excluded from the target-ROI if the wavefront did not show sufficient rotation. Phase map C shows a case where the rotor meanders outside the ROI and temporarily leaves the observation area. In this case, the wavefront rotation cannot be confirmed sufficiently. In the snapshot at 0 ms, a phase singularity (core) is observed at 8 o’clock in the boundary region of the ROI. The rotor meandering toward the boundary of the ROI. In the snapshots from 64 ms to 128 ms, the wavefront is observed rotating counterclockwise, but the phase singularity (core) is meandering outside the ROI, and the wavefront cannot be observed thereafter. In such cases, the peripheral area is mapped and evaluated. By closely observing the wavefront dynamics of the neighboring ROI, the rotor was identified, and its stability was evaluated. Phase map D shows snapshots of the ROI where a stable rotor was identified, which was determined as the “target ROI.” In the 0 ms snapshot, the phase singularity (core) is observed at the 6 o’clock position. The wavefront associated with this is observed at the 8 o’clock position. After that, the rotor meanders in the direction of 12 o’clock, and the wavefront rotates counterclockwise. In the 160 ms snapshot, the wavefront has rotated 360 degrees (1 cycle) and continues to rotate. The ROI in which the rotor was observed to exhibit such stable wavefront dynamics was determined to be the “target ROI,” and the rotor was targeted for low-power ablation.

This study introduces a novel approach to RM, integrating low-power RF ablation targeting visually identified rotors with PVI using cryoballoon (Table 2). The RM procedure was performed at our center on patients diagnosed with PeAF and long-standing PeAF who had not responded to anti-arrhythmic therapy. The RM group included 50 consecutive patients who underwent ablation procedures beginning in 2020. The study was approved by the institutional ethics committees, complied with the principles of the Declaration of Helsinki, and obtained written informed consent from all patients. A contrast-enhanced computed tomography (CT) scan was performed within 1 week before the procedure to assess anatomical structures. Atrial volumes were measured using diagnostic imaging software (Zio Station 2, Amin Inc., Tokyo, Japan; Figure 1). All patients received anti-coagulation therapy, and transesophageal echocardiography was conducted to exclude the presence of intra-cardiac thrombi. The procedure was performed primarily under intravenous anesthesia with dexmedetomidine or propofol. However, for 3 patients with severe sleep apnea syndrome, the procedure was performed under general anesthesia. During the procedure, intravenous heparin was administered to maintain an activated clotting time (ACT) exceeding 350 seconds. Intra-cardiac cardioversion and AF induction testing were performed using a 6.0 Fr, 20-pole mapping catheter with defibrillation capacity (BeeAT, Japan Lifeline, Tokyo, Japan), which was introduced via the right internal jugular vein. For vascular access, the following sheaths were inserted through the right femoral vein: 8.0 Fr long sheath (Swartz Braided Transseptal Guiding Introducers, St. Jude Medical Inc., Saint Paul, NM) for transseptal puncture and mapping catheter insertion. The 10.0 Fr long sheath (Swartz) was used for the intra-cardiac echocardiography catheter (ViewFlex, St. Jude Medical). A 6.0 Fr sheath was used for the right ventricular pacing electrode. Following a single transseptal puncture, bi-atrial electro-anatomical mapping and 3-dimensional geometry reconstruction were performed using the EnSite NavX mapping system (Abbott, Abbott Park, IL). Phase mapping was subsequently conducted in the LA using a 20-pole, spiral-shaped electrode catheter (Reflexion HD, St. Jude Medical), which had a diameter of 2.5 cm and a surface area of 4.91 cm^2^. For real-time phase mapping, the ExTRa mapping system (Nihon-Kohden Co., Ltd., Tokyo, Japan) was integrated with the EnSite NavX system (Figure 2). The ExTRa mapping system (EXT) processed bipolar signals computationally, using a total of 41 signals, which included individual recording signals from a 20-pole spiral catheter as well as virtual signals derived from peripheral signal calculations.^11^ The system has the capacity to record electrical activity within the region of interest (ROI) for a user-defined observation period of 5 to 8 seconds, which may enable real-time phase mapping. It appears to automatically detect phase singularities (core) and wavefronts and classify them into distinct patterns. Rotor or single rotations and multiple wavelets movements are displayed separately as “R” and “M.” The proportion of these electrical activities is quantified as the percentage of non-passive activation (%NP) during the observation period. In instances where neither of these activities is detected, the system determines the percentage of passive activation (Figures 3 and 4). The EXT system also generates high-density color phase movies in real time, allowing operators to visually assess wavefront rotation and the meandering phenomenon at one-tenth the normal speed. It is important to note that previous clinical studies using the EXT system have employed %NP as a treatment indicator. In this study, while the %NP was referenced, the primary focus was on wavefront dynamics observed in color phase movies. For rotor identification, only spiral waves that complete at least one full 360-degree rotation (1 cycle) were classified as sustaining rotors capable of maintaining AF. This classification was determined by visual analysis of color phase movies. The contact mapping system used in EXT offers high spatial resolution. However, unlike basket catheters, its field of view is limited. Therefore, it is important to carefully observation of the mapping area to establish a relationship between its size and the location of wavefront dynamics. If phase singularities or wavefronts were observed at the periphery of the ROI, remapping of the surrounding area was always performed to maintain high detection accuracy for rotor identification. An example of wavefront dynamics captured in a color phase movie is presented in Figure 5. Using this method, the ROI containing the rotor responsible for sustaining AF was designated as the “target-ROI” for low-power RF ablation. Wavefront dynamics were first analyzed in the LA, followed by the right atrium (RA). Once the target ROIs were identified in both atria, PVI was performed using a cryoballoon (Arctic Front, Medtronic, MN). Following PVI, intra-cardiac cardioversion was conducted to restore sinus rhythm. In patients who successfully converted to sinus rhythm, 10 to 20 mg of adenosine triphosphate were injected rapidly under isoproterenol infusion to assess dormant conduction. Additionally, a continuous isoproterenol infusion was administered to facilitate AF induction via burst pacing. The isoproterenol protocol included the following: Initial bolus injection of 10 micrograms, followed by a continuous infusion of 1.0 to 2.0 μg per minute, target heart rate increase of at least 30% above baseline, Systolic blood pressure maintained above 60 mm Hg. The AF induction was performed using burst pacing, which involved the use of 20 mA, 1.0 ms pulse duration, an initial pacing cycle length (PCL) of 300 ms, which was progressively shortened by 10 ms a time until the PCL reached 150 ms, or 2 :1 atrial response was observed, subsequent 60-second burst pacing was applied at the distal coronary sinus and the RA, at the shortest pacing cycle length closest to the refractory period. AF episodes lasting more than 3 minutes were classified as a positive induction. Generally, the definition of AF is a duration of 30 seconds or more. However, in our AF induction method, we observed that in many cases, AF spontaneously terminated within 3 minutes of exceeding 30 seconds. Therefore, in this study, the positive criterion was set at 3 minutes. In these cases, an attempt was made to restore sinus rhythm via cardioversion. At each stage of treatment, AF induction testing and cardioversion were conducted to assess the effectiveness of therapy. Following these assessments, the RM of the LA was performed. The target ROIs were ablated using an open-irrigated RF ablation catheter with contact force sensing (TactiCath SE, Abbott, Abbott Park, IL). RF energy was delivered at 25 watts (W) for 15 seconds per point. We made a point of maintaining the lesion size index at ≤ 3.0 to control the extent of energy application. The ablation was performed point-by-point and uniformly across all target ROIs. It is important to note that areas around the PV that had already been ablated via cryoballoon were excluded from the RM. Following LA RM, AF induction testing and cardioversion were repeated. Once LA modification was complete, RM of the RA was performed. To avoid complications, target ROIs near the sinus node or atrioventricular node were excluded from low-power ablation to avoid complications. The inferior RA region was also excluded. These regions contain anatomical structures (Eustachian valve, Thebesian valve, and Chiari network) that have the potential to induce conduction delays. Therefore, linear RF ablation, which is an ablation procedure for common AFL as an alternative to low-power ablation, was performed. Following RA modification, AF induction testing and cardioversion were conducted once again. The RM protocol was performed in the following order: PVI, LA modification, and RA modification. Between each treatment step, AF induction tests and cardioversions were conducted. These tests were conducted to assess the effectiveness of each ablation procedure step. However, In the case with non-PV focus, AT, or AFL was induced, and RF ablation was performed immediately in each instance.

**Table 2:** Procedural workflow of two ablation procedures

AFL = atrial flutter; AT = atrial tachycardia; ATP = adenosine triphosphate; CS = distal site of coronary sinus; ISP = isoproterenol; LA = left atrium; PV = pulmonary vein; PWI = posterior wall isolation; RA = right atrium; RF = radiofrequency; RM = rotor modification.

### Posterior Wall Isolation

**Figure 6:**
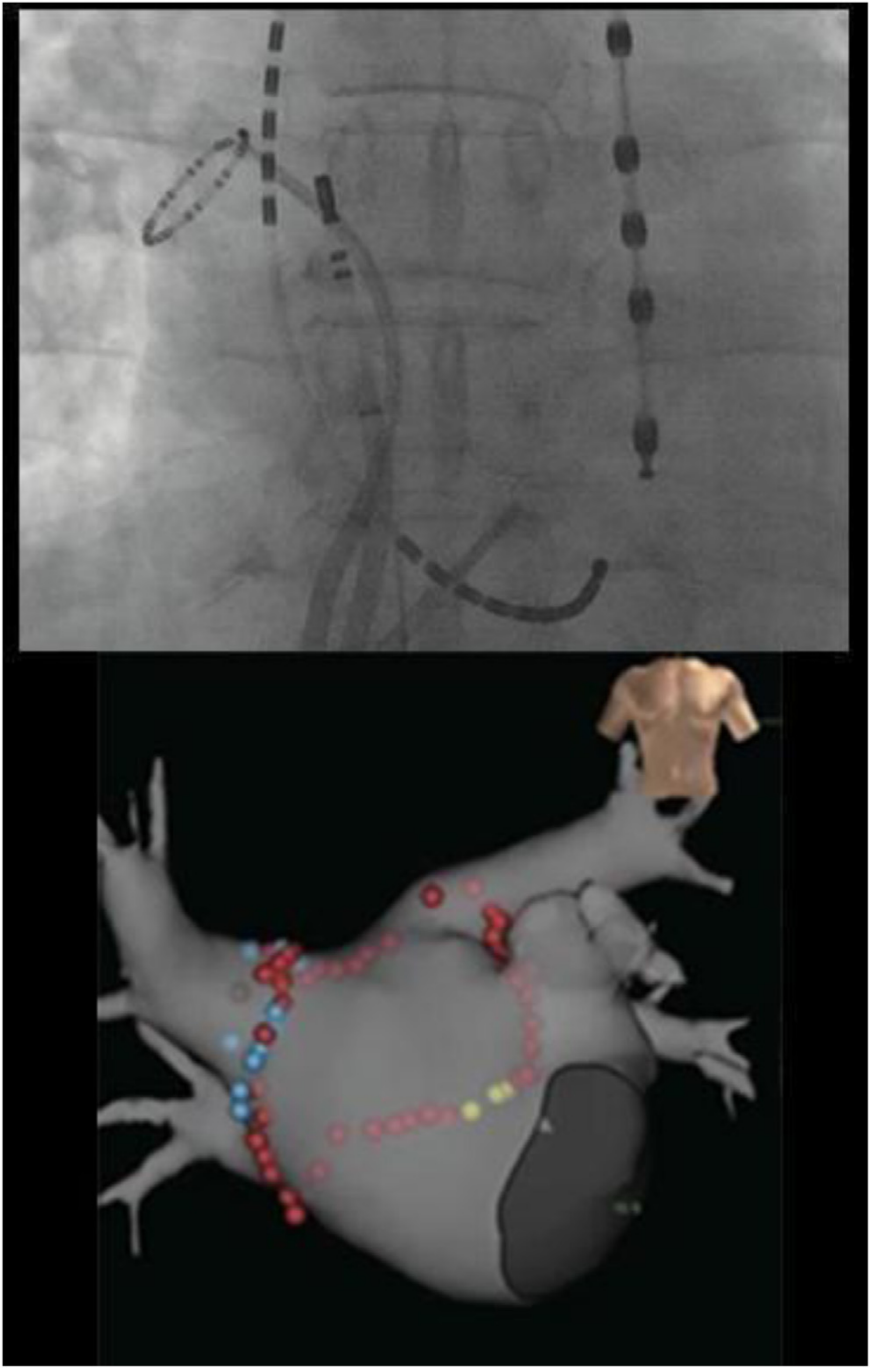
The figure demonstrates an example of the posterior wall isolation procedure. The upper image shows a frontal fluoroscopic view, displaying a ring electrode catheter and a radiofrequency ablation catheter placed in the right pulmonary vein. The lower image shows a 3-dimensional geometry model created by EnSite NavX fusing images. *Red tags* represent the ablation points. *Yellow tags* indicate ablation points where power was limited to protect the esophagus. *Blue tags* show additional ablation points for gaps in the ablation line.

PWI was performed using the BOX isolation method^4^^,^^5^ which involves comprehensive isolation of the posterior LA, including all PVs. Intravenous sedation was administered to most patients using dexmedetomidine or propofol. Six patients with severe sleep apnea syndrome underwent the procedure under general anesthesia. Mapping and navigation were performed using EnSite NavX (Figure 6). The AF induction testing, cardioversion protocol, and the isoproterenol dosage followed the same methodology as in the RM group (Table 2). Intra-cardiac cardioversion and AF induction testing were conducted using a 6.0 Fr, 20-pole mapping catheter with defibrillation capacity (BeeAT), introduced via the right internal jugular vein. The following sheaths and catheters were placed via femoral access. Right femoral vein: 8.0 Fr long sheath (Swartz) for transseptal puncture and mapping catheter insertion, 8.0 Fr long sheath (Swartz) for RF ablation catheter, 6.0 Fr sheath for right ventricular pacing electrode. For the left femoral vein, the following were used: 10.0 Fr long sheath (Swartz) for intra-cardiac echocardiography catheter (ViewFlex, St. Jude Medical). After performing a double transseptal puncture, the LA geometry was reconstructed using a circular electrode catheter (Optima, Abbott, Abbott Park, IL). The roof line was created using linear ablation from the top of the left ridge to the top of the right superior PV. The bottom line was formed by linear ablation extending from the bottom edge of the left inferior PV to the bottom edge of the right inferior PV. This approach was chosen to electrically isolate not only the PVs but also the arrhythmogenic substrates surrounding the PVs and posterior wall, which as known to contribute to AF recurrence. Antral PVs and the posterior wall were isolated using a point-by-point technique with an open-irrigated RF ablation catheter (TactiCath SE). RF energy was applied for 30 to 35 seconds at 30 to 35 W or for 3 seconds at 50 W for targeted lesion creation. In the vicinity of the esophagus, the RF energy was kept to a maximum of 20 W to reduce the risk of thermal injury. A single linear ablation lesion was created under the image guidance of imaging, using the EnSite NavX “fusion” mode, which integrates contrast-enhanced-CT scan data with the electro-anatomical mapping model for precise lesion placement. Following linear ablation, a pacing study was performed under isoproterenol infusion to evaluate the integrity of the isolation line. The entrance and exit blocks were identified, and additional RF ablation was performed on the gap.

### Statistical analysis

The comparison between the RM group and the PWI group was evaluated by analyzing the event-free survival rate, with freedom from AT/AF/AFL designated as the primary end point. The comparison of the survival curves of the 2 groups was performed using the log-rank test. The detection of AT/AF/AFL was performed by analyzing 12-lead ECGs and 24-hour Holter ECGs. Recurrence of AF was defined as an episode lasting at least 3 minutes, and AT and AFL were defined as an episode lasting at least 30 seconds. For short-term efficacy in both groups, the procedure time (in minutes) and complication rates were compared. In the RM group, the characteristics of the LA and right atrial rotors were compared. The number of detected target ROIs, the number of ablated target ROIs, the rotor cycle, and atrial volume were compared between the left and right atria. The distribution of target ROIs identified by the rotor was also investigated. In addition, the atrial volume of the RM group with PeAF cases was compared with those of paroxysmal AF (control group). Continuous variables that followed a normal distribution were expressed as mean ± standard deviation and continuous variables not following a normal distribution were presented as median and quartiles. For comparisons between the two groups either a t-test or Wilcoxon’s rank-sum test was applied, depending on the data distribution. A *P* value of <.05 was considered statistically significant. All statistical analyses were conducted using R software (version 4.3.2).

## Results

**Figure 7:**
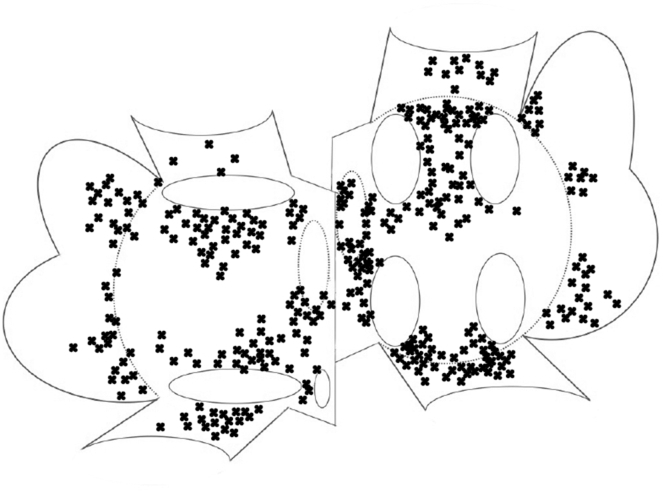
This schematic diagram shows the distribution of the locations identified as “target-ROI” based on the classification of wavefront dynamics. The “target ROIs” are indicated by “X” marks in the schematic diagrams of both atria. Each region is labeled with its anatomical terms. The atrial septum is shown from both sides, indicating the locations recorded in LA mapping and right atrial mapping, respectively. The “X” mark indicates the central position of the “target-ROI.” The area of the ROI is 4.3 cm^2^, and the phase singularity is meandering, the position does not exactly match the “X” position.

**Figure 8:**
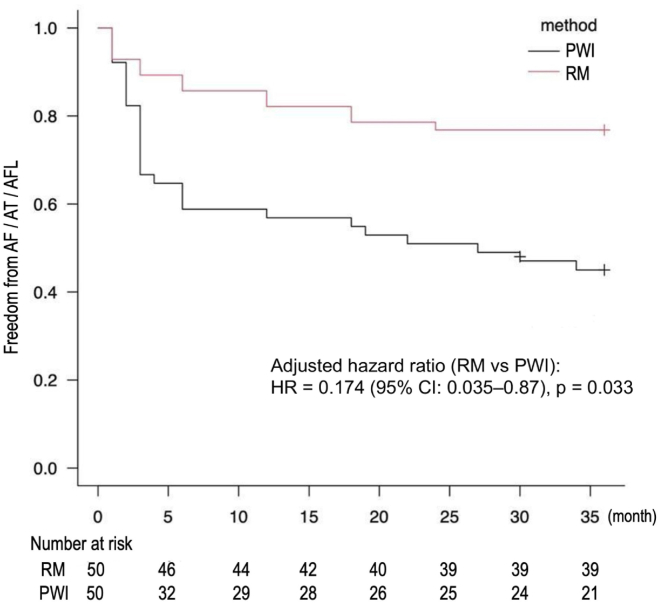
This figure shows the survival curves analyzed using the Kaplan-Meier method. The analysis was based on the event-free survival rate over a 36-month observation period, and recurrence of atrial tachycardia (AT), atrial fibrillation (AF), and atrial flutter (AFL) was defined as the end point. The log-rank test was used to analyze the survival curves, and the *P* value was <.05, indicating a statistically significant difference. The number of patients evaluated at each time point is presented in the graph below. The following table shows the data for the duration of the procedure and the number of complications in both groups.

In the RM group, phase mapping was performed on an average of 20.9 ± 5.3 sites in the LA and 19.3 ± 3.9 sites in the RA. The average time required for phase mapping was 6.4 ± 2.9 minutes in the LA and 5.9 ± 1.5 minutes in the RA. The number of target ROIs detected using wavefront dynamics was 4.0 ± 1.7 in the LA and 3.8 ± 1.5 in the RA. The average cycle of the rotor was 167.9 ± 22.5 ms in the LA and 164.8 ± 33.4 ms in the RA. The number of ablated target ROIs, into account the excluded regions, was 3.2 ± 0.5 in LA and 3.0 ± 0.3 in RA. In the RM group, the LA volume index was 74.3 ± 18.8 mL/BSA, and the RA volume index was 78.4 ± 29.6 mL/BSA, and no significant difference was observed between the 2 atria (Table 3). The distribution plot in which the target-ROI was detected is shown in Figure 7. The site indicated by “X” represents the center of the site where the target-ROI was detected. It was observed that the distribution of the target-ROI was not uniform in both atria and was concentrated around anatomical structures. The outcomes of AF induction tests and cardioversion performed after each procedure step in RM protocol are summarized in Table 4. Following the final treatment step of RM (RA modification), AF could no longer be in 45 out of 50 cases (Table 4). The efficacy of RM was evaluated by comparing it with PWI. The Kaplan-Meier curve was used for the statistical analysis of differences between the treatment groups. In the 36-month follow-up, the freedom from AT/AF/AFL was 76.8% in the RM group and 45.0% in the PWI group. The log-rank test was used to analyze the survival curve, and the *P* value was less than .001 (Figure 8). The multivariable Cox regression analysis showed that the RM was associated with a significantly lower risk of recurrence compared to the PWI (hazard ratio [HR], 0.174; 95% confidence interval, 0.035–0.87; *P* = .033). Other covariates including age (>65 years), female sex, body mass index (>25 kg/m^2^), left ventricular ejecton fraction(<55%), and treatment year, were not statistically significant. The overall model showed good fit (C-index = 0.68) (Table 5). There was no significant difference in complication rates between the 2 groups. In the RM group, there were 2 cases of vascular complications (hematoma) and 1 case of false femoral aneurysm, while in the PWI group, there were 2 cases of vascular complications (hematoma) and 1 case of tamponade (Table 1).

**Table 3:** Comparison of rotor characteristics and mapping parameters between the right and left atria

	RA	LA	*P* value
Volume/BSA, mean (SD), mL/m^2^	78.4 (29.6)	74.3 (18.6)	.730
Mapping time, mean (SD), min	5.9 (1.5)	6.4 (2.9)	.625
No. of observed ROI, mean (SD), pts	19.3 (3.9)	20.9 (5.3)	.800
No. of detected target-ROI, mean (SD), pts	3.8 (1.5)	4.0 (1.7)	.916
No. of ablated target-ROI, mean (SD), pts	3.0 (0.3)	3.2 (0.5)	.629
Rotor cycle, mean (SD), msec	164.8 (33.4)	167.9 (22.5)	.799
No. of rotation of wavefront, median (25, 75%)	2.1 (1.0, 12.2)	2.2 (1.0, 13.0)	.938

Significant difference at < .05.

BSA = body surface area; LA = left atrium; RA = right atrium; ROI = region of interest.

**Table 4:** Termination rate of atrial fibrillation after each step of Rotor Modificationsてp

	No. of cases	No. of cases	No. of cases
	Returned to SR	AF not induced	AF termination during ablation
Strategy			
Phase mapping			
Cardioversion	40 cases		
PV isolation			0
Cardioversion	42		
AF induction test		33	
LA rotor modification			2
Cardioversion	44		
AF induction test		36	
RA rotor modification			2
Cardioversion	49		
AF induction test		45	
Cardioversion	50		
			(number of cases/50)

AF = atrial fibrilliation; LA = left atrium; PV = pulmonary vein; RA = right atrium; SR = sinus rhythm.

**Table 5:** Cox proportional hazards model assesing the effectiveness of Rotor Modification vs. Posterior Wall Isolation

	HR	95% CI	*P* value
RM	0.174	0.035–0.866	.033
age (> 65 years)	1.73	0.682–4.37	.249
gender (female)	1.18	0.518–2.74	.681
BMI (>25)	0.76	0.359–1.61	.474
EF (<55)	1.25	0.551–2.83	.595
year (procedure)	1.60	0.794–3.23	.188

BMI = body mass index; CI = confidence interval; EF = ejection fraction; HR = hazard ratio; RM = rotor modification.

## Discussion

Various ablation techniques have been developed for managing PeAF, with PVI and PWI being among the most widely used. Over time, PWI has evolved into several methods, including the BOX isolation method,^5^ cryoballoon-assisted posterior wall ablation or isolation method,^6^ and hybrid convergent procedure.^7^ At our center, we have performed BOX isolation as a treatment for PeAF. However, in cases with marked atrial enlargement, the procedure has presented certain challenges, and the recurrence rate of AF has not been negligible. Consequently, we sought to identify a more effective treatment method. Among the various treatment methods, we focused on RM. In patients who experienced AF recurrence after undergoing PWI, we used the EXT system^11^^,^^19^, ^20^, ^21^ and detected spiral wave activity originating from areas outside the isolated regions of PWI, including the RA. Ablation targeting these rotors successfully restored sinus rhythm in many of these patients. Based on this experience, we initiated this study to assess the effectiveness of RM as an independent ablation strategy.

It is widely accepted that the mechanisms of AF involve both random reentry and spiral reentry.^22^, ^23^, ^24^, ^25^ Multielectrode mapping^26^^,^^27^ and optical mapping^28^^,^^29^ studies have confirmed the presence of multiple wavelets, wave breaks, and meandering conduction patterns. Clinical evidence suggests that stable rotors located in either atrium can sustain complex conduction, particularly when driven by high-frequency rotation. When multiple rotors are present, the one with the highest frequency often dominates atrial activation.

The Conventional Ablation for Atrial Fibrillation With of Without Focal Impulse and Rotor Modulation (CONFIRM) trial was the first to demonstrate that targeting such stable rotors and focal sources using phase mapping could effectively sustain sinus rhythm.^10^ The EXT system used in our study provides real-time visualization of atrial electrical activity during AF and allows for the identification of rotor cores. Its performance has been validated in experimental models, where it showed a strong correlation with optical mapping,^30^ particularly in capturing spiral reentry. The system quantifies the percentage of NP activation around phase singularities. However, this metric includes both sustained rotors and transient small wavefronts. To differentiate the two, we analyzed color-coded phase movies to visually assess wavefront behavior and selected only those regions showing sustained rotational activity. Interestingly, these rotors were not evenly distributed and occurred with comparable frequency in both atria.

During Focal Impulse and Rotor Modulation (FIRM)-guided ablation, an average of 2.2 ± 1.0 stable AF sources per patient were identified, with 76 % located in the LA and 24 % in the RA.^10^^,^^12^ Noninvasive mapping studies have reported that recurrent reentry and single-rotation activity accounted for 73 ± 11 % and 27 ± 11 % respectively, with a median of 2.6 rotations.^15^ In contrast, our data showed an average of 4.0 ± 1.7 rotors in the LA and 3.8 ± 1.5 in the RA. Several factors may explain this discrepancy, most notably the differences in catheter technology. The FIRM system uses a non-contact basket catheter that provides panoramic atrial coverage but lacks spatial resolution at the poles. In comparison, the contact-mapping-based EXT system offers higher local resolution but is limited by a smaller mapping area, preventing full atrial assessment in 1 session. These differences likely affected rotor detection outcomes.

We also found that most rotors detected by EXT were situated near specific anatomical structures. In the LA, rotors were frequently detected around the PVs and atrial septum, while in the RA, they were commonly observed near the posterior walls of the superior and inferior vena cava. Anatomical structures such as the pectinate muscles, crista terminalis, Bachmann’s bundle, PV ostia, coronary sinus, and ligament of Marshall are thought to contribute to conduction delay and serve as anchoring sites for rotors.^31^, ^32^, ^33^ Notably, regions such as the vena cava–atrial junctions and the fossa ovalis—with its complex embryological layering—may promote reentry. Additionally, the inferior posterior wall of the LA, which lacks pericardial inversion, may be prone to mechanical stretching. These areas show significant heterogeneity in wall thickness and fiber orientation, factors that may facilitate rotor stabilization.^34^^,^^35^

The posterior wall of the LA has traditionally been considered a critical target in AF ablation. Previous studies have emphasized the role of Bachmann’s bundle and multiple conduction pathways in maintaining AF. However, our mapping showed that rotors were rarely observed in the LA posterior wall, possibly due to reduced mapping sensitivity caused by compression from the vertebrae. On the other hand, contrast-enhanced CT in our study revealed marked enlargement of both the LA and RA in PeAF patients. Rotor characteristics did not significantly differ between the LA and RA. These findings support the rationale for a bi-atrial ablation approach and suggest that targeting both atria may enhance the efficacy of substrate modification in PeAF.

## Conclusion

The study investigated the potential benefits of RM for PeAF in comparison to PWI. The results of volume and phase mapping for both atria were comparable, suggesting the potential importance of the approach to the RA. The results of RM were encouraging in terms of reducing the long-term recurrence rate. It is important to note that this study was conducted at a single institution and used before-and-after comparison design. To determine the effectiveness of the treatment approach of low-power ablation to a single meandering rotor in both atria, it is necessary to investigate it in a larger-scale clinical trial.

## Limitations

The study was designed as a non-randomized, before-and-after cohort study, with differing enrolment periods for the PWI and RM groups. As a result, temporal advancements in supportive care or procedural techniques may have affected clinical outcomes independently of the ablation strategy itself. Furthermore, variations in the PVI protocol applied in the RM and PWI groups may have contributed to differences in long-term outcomes. In the RM group, phase mapping was performed only once for 3 seconds at each site, without subsequent remapping. Consequently, changes in rotor location after AF recurrence were not assessed. In addition, simultaneous mapping of both atria was not performed, limiting the ability to evaluate atrial activation in a comprehensive manner. The definition of AF recurrence used in this study (an episode lasting at least 3 minutes) deviates from widely accepted criteria, which may have influenced the interpretation of recurrence rates.
